# Enhanced Mechanical Performance of Bio-Inspired Hybrid Structures Utilising Topological Interlocking Geometry

**DOI:** 10.1038/srep26706

**Published:** 2016-05-24

**Authors:** Lee Djumas, Andrey Molotnikov, George P. Simon, Yuri Estrin

**Affiliations:** 1 Department of Materials Science and Engineering Monash University, Victoria, 3800, Australia; 2 Laboratory of Hybrid Nanostructured Materials, NUST MISiS, Moscow 119490 Russia

## Abstract

Structural composites inspired by nacre have emerged as prime exemplars for guiding materials design of fracture-resistant, rigid hybrid materials. The intricate microstructure of nacre, which combines a hard majority phase with a small fraction of a soft phase, achieves superior mechanical properties compared to its constituents and has generated much interest. However, replicating the hierarchical microstructure of nacre is very challenging, not to mention improving it. In this article, we propose to alter the geometry of the hard building blocks by introducing the concept of topological interlocking. This design principle has previously been shown to provide an inherently brittle material with a remarkable flexural compliance. We now demonstrate that by combining the basic architecture of nacre with *topological interlocking* of discrete hard building blocks, hybrid materials of a new type can be produced. By adding a soft phase at the interfaces between topologically interlocked blocks in a single-build additive manufacturing process, further improvement of mechanical properties is achieved. The design of these fabricated hybrid structures has been guided by computational work elucidating the effect of various geometries. To our knowledge, this is the first reported study that combines the advantages of nacre-inspired structures with the benefits of topological interlocking.

Many natural or biological structural materials are composites or hybrid structures^1^ with outstanding mechanical properties, which, surprisingly, are often achieved due to the presence of relatively weak constituents^2^. The resultant materials are also remarkable with regard to the efficient use of available resources, made possible by the process of evolution. Indeed, many biological systems have mechanical properties that far exceed those of man-made materials^3^ and often have served as an inspiration for materials engineers in their quest for novel materials^4^,^5^,^6^,^7^,^8^,^9^,^10^. Some now “classic” examples of structural natural materials include wood^11^, bamboo^12^, bone^13^, and shells of some molluscs^14^.

The key to the outstanding performance of these biological materials is believed to be in the shape and spatial arrangement of their constituents and the hierarchical nature of these composites^15^. In particular, it is the complex hierarchical architecture these materials possess across multiple length scales that gives rise to the exceptional functionalities observed in Nature. One such example is nacre, the iridescent mother of pearl, found on the inner shell of some molluscs, which has attracted much interest from materials scientists due to its exceptional mechanical properties^16^,^17^,^18^. While nacre is made up nearly entirely of aragonite, a highly brittle ceramic, it displays a unique combination of stiffness, strength, fracture toughness and energy absorption capability. At the core of this behaviour is the microarchitecture of nacre: a brick-and-mortar like structure made up of polygonal aragonite platelets forming a lamellar structure (95 wt%) with a soft, viscoelastic organic protein (5 wt%) binding the lamellae together^19^.

Despite the preponderance of aragonite in its structure, nacre is 3000 times tougher than monolithic aragonite^20^, indicating that this behaviour is due to nacre’s composite nature and architecture, rather than the properties of the majority constituent. Over the last decade, various structural features were identified as contributors to the toughness of nacre, including nano-asperities and the microscale waviness of the platelets, among others^16^,^21^,^22^,^23^,^24^. What has transpired from these studies is that the *spatial arrangement* of the elements of the nacre structure is crucial for its outstanding mechanical properties. Under load, these structures generate progressive locking of slip between the adjacent platelets, causing hardening and toughening of the structure^23^,^25^,^26^. Many examples of mimicking various aspects of the nacre structure (and that of other natural materials) in synthesising engineered materials have been reported^9^,^17^,^19^,^27^,^28^,^29^,^30^,^31^,^32^, mostly with very encouraging results. In this work, we will combine the materials design concepts inspired by nacre-like structures with the relatively new concept of topological interlocking, which broadens the design space.

*Topological interlocking*^33^,^34^,^35^,^36^,^37^,^38^,^39^,^40^,^41^,^42^,^43^,^44^,^45^ has been suggested as a novel method to create architectured materials. This concept relies on segmenting a monolithic structure into specifically shaped elementary blocks, which are constrained in their movement by the neighbouring elements. It is a distinctive feature of topological interlocking that not only in-plane, but also out-of-plane movement of a block is hindered by its neighbours. As a consequence, no connectors or binders are needed to hold the blocks in place. These assemblies only require a global constraint, such as an external frame, tensioned wires passing through the blocks, or corner fasteners, to maintain the integrity of the overall structure. Multiple block geometries that permit topological interlocking have been identified^38^.

Dyskin, Estrin and colleagues have shown that certain convex polyhedra, notably all five platonic bodies (tetrahedron, cube, octahedron, dodecahedron, and icosahedron), as well as their derivatives, allow for topological interlocking^33^,^34^,^46^. Significant studies on plates assembled from tetrahedron-shaped elements have been conducted^47^,^48^,^49^, with stiffness-scaling relationships established^50^ and thrust line models developed to describe mechanical behaviour of such assemblies^51^. This concept has been further developed to include building blocks with non-planar surfaces^52^. These interlockable blocks have specially engineered geometries with matching concavo-convex faces. They are referred to as *osteomorphic* due to their similarity to the shape of a bone (Fig. 1d). This shape was developed mathematically and must meet certain periodicity and symmetry requirements; one such function describing the curved edges of a block is the cosine function (with a 90° shift of the argument at the opposite edge), which is used in this work.

Topological interlocking, while providing structural stability, allows for some restricted movement of the elements relative to one another. This ensures that the structure is more compliant than a monolithic one, and is also able to absorb vibrational energy, which is dissipated by frictional losses^39^. As a clear demonstration of the tolerance of topologically interlocked materials to local failures, Molotnikov *et al*.^36^ showed that they are able to maintain structural integrity until as many as approximately 25% of the elements fail at random. Indeed, the resistance of the assemblies to removal or displacement of an element is enhanced due to the opposite inclinations of the surfaces of neighbouring blocks, while escalation of local damage to a catastrophic crack does not occur, as a crack developing within a block cannot propagate beyond its boundary.

The concept of segmenting a monolithic structure into a set of discrete topologically interlocked elements represents a highly promising avenue for structural materials design. Whilst the idea of segmentation is inspired by the biological materials, the concept of topological interlocking is not directly motivated by Nature, nor has it been observed in living organisms. The closest analogy found in Nature is the suture of red-eared slider turtle^53^, however its interdigitated features are not of the kind discussed in the context of osteomorphic or polyhedral-based interlocking assemblies. It has been observed^54^ that natural systems have evolved to meet highly specific “design criteria”, as given by their distinct and often unique conditions. As such, understanding and utilising the *structural mechanisms* of these systems should prove more rewarding for materials research than direct *replication* of these systems using synthetic constituents. It is within this context that the opportunity to explore the potential of improving designs found in Nature by augmenting it with the artificial topological interlocking structures appears exciting.

In this work, we combine the principle of topological interlocking with the concept of bio-inspired design and realise the synthetic structures thus conceived by employing 3D printing technology. This permits the integration of multiple materials with widely contrasting mechanical properties within a hybrid structure in a single build. Previous work in this area has primarily focussed on structures for which the shape of the individual elements did not vary in the direction normal to the plane of assembly, essentially two dimensional studies^55^,^56^,^57^,^58^,^59^. The present work extends these concepts by using building blocks with more complex geometries, allowing for shape variation in the direction normal to that plane. This *three-dimensional* osteomorphic block geometry offers itself as a basis for such design, as the mentioned benefits of topological interlocking may add to the attractive properties of natural nacre. In this connection, it should be mentioned that a tessellation of a massive plate into a set of osteomorphic blocks resembles the pattern in which aragonite platelets are arranged in nacre. Furthermore, the natural surface waviness of the aragonite platelets that creates a simple form of mechanical locking is ‘amplified’ in the concavo-convex contact surfaces in an ensemble of osteomorphic blocks. Below we report the results of experimental investigations of the mechanical behaviour of the novel topological interlocking structures fabricated by additive manufacturing. Also presented are the outcomes of computational work carried out to support the experiments and elucidate the mechanical response of the nacre-inspired structures with topologically interlocked hard building blocks.

## Results

**Figure 1:**
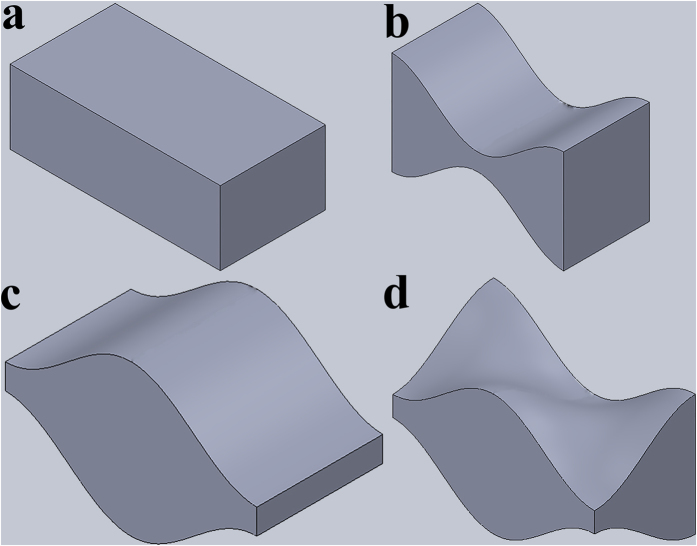
Principal building block geometries used in specimens for testing. (**a**) rectangle or standard brick used as a reference in the brick-and-mortar structures printed; (**b**) “hourglass” and (**c**) “smooth honeycomb” represent the two opposite faces of the osteomorphic block shown in (**d**). The size of each element pictured is 6.25 mm in length and 3.125 mm in width, however the physical dimensions could be chosen arbitrarily, as the property of interlocking is size-independent. Note that the cross-sections of the blocks in figures (**b**,**c**) do not change along the directions normal to the faces. Assemblies based on these geometries are therefore referred to as two-dimensional. By contrast, the cross-section of the osteomorphic block (**d**) transforms along the direction normal to its face: a crest at the front face corresponds to a minimum at the back face. The shape presented in (**d**) provides the structure with the three-dimensionality that is crucial for its mechanical performance.

Four principal geometries were selected for testing (Fig. 1). The brick-and-mortar design (Fig. 1a) emulating nacre was used as a reference due to recent interest it has garnered^26^. However, the main focus of this study was on the effect of three-dimensional interlocking offered by the *osteomorphic* design (Fig. 1d). In order to verify the ability of 3D design of a building block to enhance mechanical properties of the assembly, two other types of block shapes were also developed, based on the surface profiles of the two opposing faces of the osteomorphic block. These modified blocks have the same thickness and geometry of the curved contact surfaces as the osteomorphic blocks, but vary from them in terms of width. The two variants considered are termed “hourglass” (Fig. 1b) and “smooth honeycomb” (Fig. 1c). The dimensions of the building blocks were kept constant across specimens, with an aspect ratio of approximately 2.9. It is important to note that the dimensions selected do not reflect the aspect ratios and stiff-to-soft volume fraction ratios found in nacre, nor are they the focus of this work. While other works have addressed the optimization of these parameters^60^,^61^, the present study concentrated on the shape of the stiff blocks as a means to improve the mechanical performance of the assembly over the nacre structure seen as a reference.

**Figure 2:**
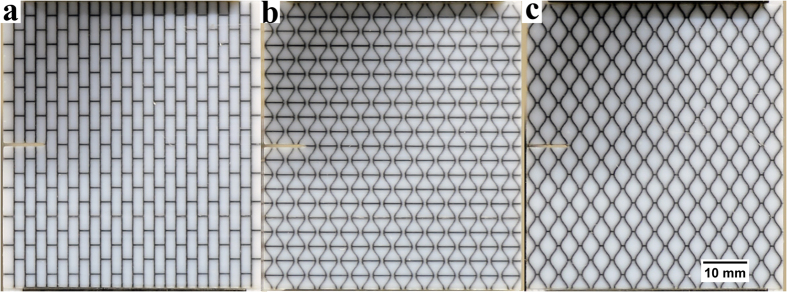
Images of additively manufactured specimens used for mode I fracture testing. The dimensions of specimens are 64.75 mm × 58.5 mm with a thickness of 3.125 mm. The volume fraction of the soft phase is approximately 13.5 ± 0.1% for all samples. (**a**) Brick-and-mortar sample, representing a simplified nacre structure; (**b**,**c**) “hourglass” and “smooth honeycomb” samples: the respective motifs of the surface tessellations of these samples correspond to the two opposite faces of an osteomorphic block, as seen in Fig. 1d. Further details of the samples are given in Methods. Note that the sample consisting of interlayered interlocked osteomorphic blocks, which was also tested, is not shown, as it appears identical to the samples in (**b**,**c**) depending on which side is viewed.

Mode I fracture tests were conducted on 3D printed specimens (Fig. 2) which combine the stiff (VW+) and the soft (TB+) materials, in a nacre-like fashion. (The acronyms VW+ and TB+ stand for the proprietary names of the Stratasys polymers VeroWhitePlus and TangoBlackPlus used in 3D printing of the structures considered.) In essence, these tests represented uniaxial tensile loading of notched specimens. The geometry of the assemblies tested was motivated by the work by Dimas *et al*. on brick-and-mortar and related two-dimensional bio-inspired structures^55^. The new element in this study was the specially designed shape of the hard blocks described above. The details of the printing process and the materials used are outlined in the Methods section.

**Figure 3:**
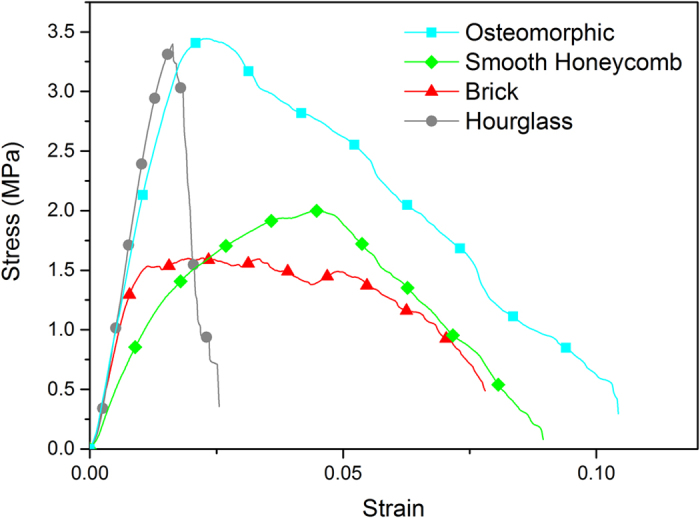
Comparison of *averaged* stress-strain curves for the four geometries. This plot illustrates the increase in peak stress, strain at failure, and modulus of toughness of the osteomorphic specimen as compared to the brick-and-mortar structure. The effect of the high peak load appears to be associated with interlocking, as it is the greatest for the “hourglass” and the “osteomorphic” cases – both involving interlocking.

Mechanical testing of these specimens returned highly advantageous results (Fig. 3). The stress-strain curves for the geometries tested demonstrate a superior mechanical performance of the osteomorphic design compared to that of the simple brick-and-mortar (Fig. 2a) or nacre-like samples (Fig. 2b,c). The hourglass geometry results also show a large increase in peak stress. It should be noted that our results for brick-and-mortar structure are in reasonable agreement with those previously reported, with an allowance for differences in the experimental procedure and the aspect ratios of stiff elements^55^. Hourglass and osteomorphic samples exhibit very similar values of peak stress. This suggests that interlocking in the direction of loading of the hourglass assembly (along the long axis of the blocks) is the predominant mechanism that leads to a high peak stress. Indeed, interlocking of the hourglass blocks in this orientation hinders slipping of the blocks in a similar (albeit not identical) way to the interlocking of osteomorphic blocks.

**Figure 4:**
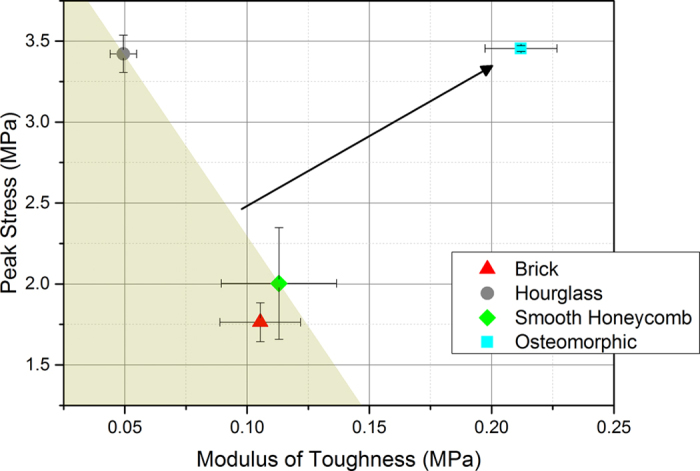
Plot of modulus of toughness (as measured by the area under the stress-strain curve) *vs.* peak stress. Doubling of both modulus of toughness and peak stress achieved with the osteomorphic geometry is seen. Improvement of both characteristic properties due to the osteomorphic design is highlighted by the arrow.

As evident from Fig. 3, the osteomorphic structure exhibits a superior performance (nearly doubling of both the peak stress and the elongation to failure) over that of the brick-and-mortar structure. Figure 4 shows a plot of modulus of toughness (as measured by the area under the stress-strain curve) *vs.* peak stress. It is seen that the assemblies made from osteomorphic blocks exhibit improvement in both modulus of toughness and peak stress over the traditional brick-and-mortar design.

**Figure 5:**
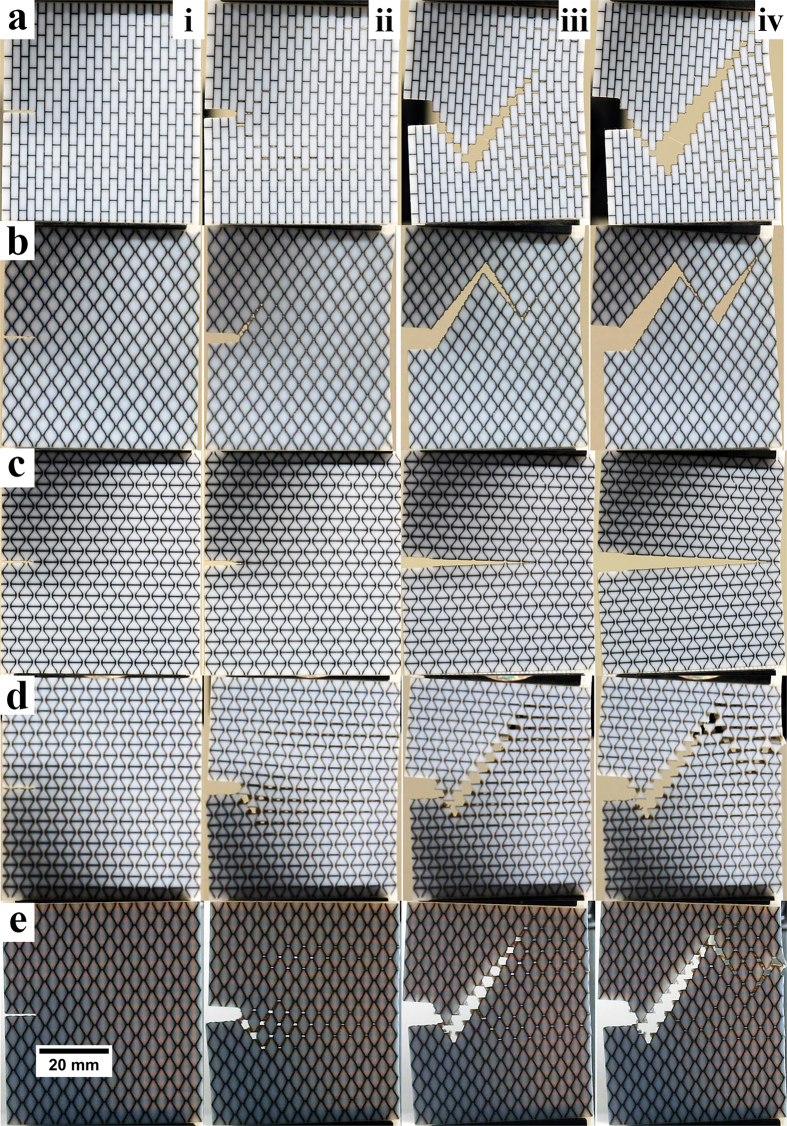
Images of fracture sequence during experimental testing for (a) brick-and-mortar, (b) smooth honeycomb, (c) hourglass, (**d**) osteomorphic – hourglass side, (e) osteomorphic – smooth honeycomb side. The images represent the four approximate stages of fracture i) initial state, ii) crack initiation, iii) crack propagation, iv) failure. Areas of stress delocalization are observed in all samples except (**c**). Of particular note are these areas observed in stage ii) of testing.Similarities in fracture path is also observed in stage iv) for all samples except (**c**).

The fracture paths in the samples tested are shown in Fig. 5. These images reveal that failure surfaces of osteomorphic, brick-and-mortar, and smooth honeycomb structures follow a similar fracture path. This suggests that the fracture path is not the sole factor in the improved modulus of toughness of the osteomorphic sample, as the mechanical responses for these designs vary significantly. These fracture patterns are relatively simple as compared to those in natural nacre, with less changes of direction or zigzagging, as one could expect from the lower aspect ratio of the building blocks in our synthesized structures.

Delocalization of stress around the crack tip was observed in all specimens (Fig. 5), except for the hourglass shape with its concave geometry. The hourglass shape is unable to provide resistance to the concentration of the tensile stress around the thin waist of a block, leading to early fracture of these blocks (stiff phase). Indeed, failure through the soft phase, rather than the interface between the soft and stiff phases (delamination), was observed for all specimens, except for the hourglass geometry.

**Figure 6:**
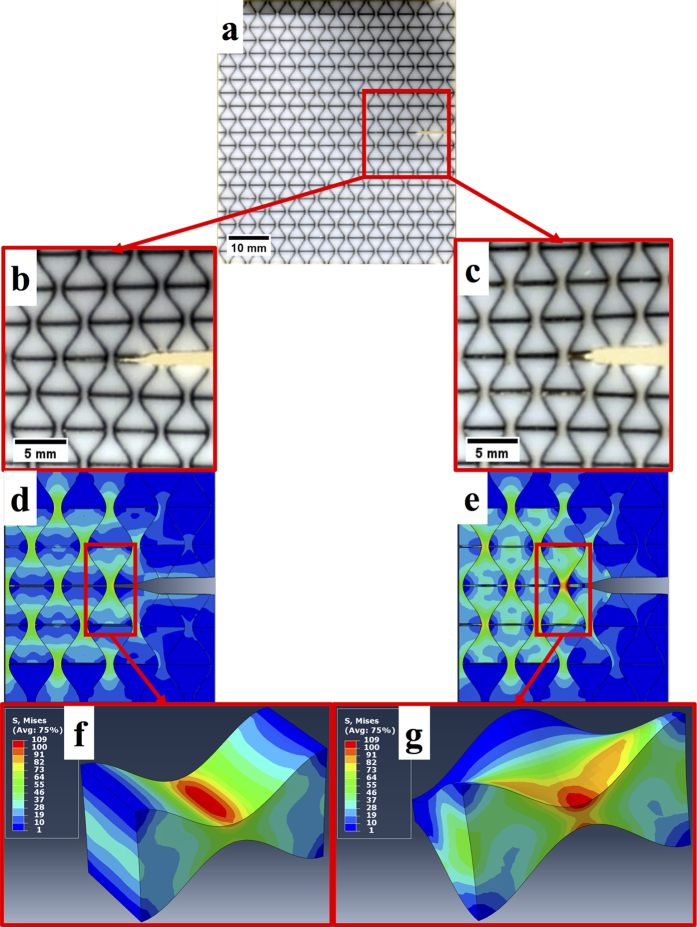
Overview of computational work: (**a**) initial state of experimental specimen with crack initiation site highlighted in red, experimental crack inititaion site of hourglass (**b**) and osteomorphic (**c**) specimens before brittle failure, FEA simulations showing von Mises equivalent stress distribution at a crack initiaion site for hourglass (**d**) and osteomorphic (**e**) specimens with central element highlighted in red, FEA simulations showing von Mises equivalent stress distribution for a central element at the crack tip for hourglass (**f**) and osteomorphic (**g**) geometries.These images illustrate the areas of stress concentration for the two geometries with interlocking features. The stress concentration in the “hourglass” block (**f**) extends through the entire depth of the specimen which results in its brittle failure. The 3D nature of the osteomorphic block (**g**) restricts the stress concentration to one side of the block, which reduces the likelihood of a crack propagation through the stiff phase.

Computational work was undertaken to gain a qualitative insight into some of the observed experimental results and the mechanisms involved. Three-dimensional finite element simulations were conducted to investigate the stress distribution in the area around the crack initiation site, as it was of particular interest for the mechanical performance of the specimens (Fig. 6). This critical area focussed on two geometries that exhibit interlocking capabilities, *viz.* hourglass and osteomorphic. The scope of this work was not to simulate the actual experimental testing precisely, but rather to gain qualitative insight into the stress distributions in ensembles of interlocked blocks at the initial stages of experimental testing, i.e. at the onset of brittle failure in the hourglass specimen. This was achieved by applying a tensile load of a magnitude similar to that load at which brittle failure occurred experimentally in the hourglass geometry (involving an approximately 1 mm displacement), to a notched planar assembly of interlocked blocks from around the crack initiation site (a region highlighted in Fig. 6a–c). The soft phase was not included in the simulations, as the primary focus of the numerical work was limited to the analysis of stress distributions around the potential crack initiation site, which led to brittle failure in hourglass assemblies but not in osteomorphic ones. The latter failed through the soft phase, as the stress concentration zone was confined to a small volume at the edge of a block, see below. Due to the lack of adequate models at this scale, particularly for the soft phase, simulation of the mechanical responses of the hybrid materials was beyond the scope of this work and will be the subject of future research. The elements were modelled as linear elastic materials with an elastic modulus E = 1.8 GPa and Poisson’s ratio ν = 0.39 as derived from previous experimental work^62^. The coefficient of friction between the blocks was set at μ = 1.0 to emulate the ‘stickiness’ effect of the soft, elastomeric matrix. The simulations did not proceed to the stage of crack propagation where the cohesive effect of the soft phase is predominant. This high coefficient of friction thus acts to approximate the initial relative movement of the stiff elements.

The distributions of the von Mises equivalent stress are presented for the element in front of the initial crack tip highlighted in Fig. 6f,g. The figure illustrates the occurrence of stress concentration around the central region of the elements. For the same level of displacement of the loading frame, the maximum stresses that develop in the hourglass block (Fig. 6f) and in an osteomorphic block (Fig. 6g) are found to be nearly identical (109 MPa). However, the main difference observed between the two geometries is that the stresses propagate through the depth of the hourglass element, while the stresses in an osteomorphic block are concentrated on one side or edge of the element, cf. Figure 6. This appears to be the likely reason for greater resistance of the hard phase in the osteomorphic assembly to brittle failure, achieved by promoting failure through the soft phase. Figure 6e shows greater localisation of stress at the neck region of the osteomorphic elements compared to the hourglass ones (Fig. 6d). It is important to note that these stresses do not propagate through the depth of the osteomorphic blocks and are instead confined to a small area at the edge of the blocks, which appear to be unable to give rise to brittle failure. Further computational work to substantiate this proposition is needed. This would require the implementation of an adequate full-scale three-dimensional constitutive model with both hard and soft phases, one which accounts for damage evolution in both phases and is currently under development. Further details of the computational work can be found in the Methods section.

The designs presented in this work have a greater degree of interlocking between the hard phase blocks than that observed in nacre^26^. As mentioned above, the waviness of the aragonite platelet surfaces gives rise to a certain level of mechanical interlocking. The “waviness” of the topologically interlockable blocks is principally of a different kind. First, it is an inherent element of the structure that provides periodicity of tessellation of a plate into discrete blocks and ensures its very integrity. Second, it has a much greater amplitude than the irregular waviness of the aragonite platelets in the nacre structure. It should be noted that the magnitude of the undercut of the mating osteomorphic blocks can be varied without a change of the topology of interlocking. Our results strongly suggest that the superior performance of the osteomorphic design is related to this specialised block geometry. The curved contact surfaces of the blocks, particularly the concavo-convex surfaces of the osteomorphic ones, and the periodic nature of their arrangement result in thicker and thinner parts of the blocks facing each other at their opposite sides, helping resist premature fracture. Essentially, the advantageous effect of 2D in-plane interlocking of the dovetail kind (as found in natural nacre)^56^ or hourglass geometries is retained in 3D interlocking assemblies, with an additional benefit of interlocking in the direction *normal* to the plane of assembly. Importantly, the 3D nature of the block geometry inhibits fracture of the stiff phase and promotes failure development through the soft phase, thus enhancing the overall modulus of toughness.

Barthelat^26^ noted that control of a number of variables (including block waviness and thickness of the panel) in simple interlocked nacre-like structures was necessary to balance opposing tensile and compressive forces within stiff elements, thus hindering early fracture of the stiff phase. This brittle failure clearly should be avoided, and the 3D block geometries described in this work provide an avenue to do so. This presents new possibilities to extend the properties space of materials, due to the benefits of a high degree of interlocking (high peak stress), while “channelling” failure into the soft phase thus achieving large strains at failure.

It is also worth noting that while in Mode I fracture testing, topologically interlocked structures outperformed the more traditional ones, a similar or even higher level of improvement can be expected in other failure modes. For example, the ability of osteomorphic structures to support loads normal to the plane of assembly due to the 3D interlocking can reasonably be expected to outperform the brick-and-mortar structures, and such beneficial behaviour has already been reported in previous work by our group^42^. Further improvement of mechanical response in all modes of deformation may be possible by block geometry optimization, and this is part of our ongoing investigations.

## Conclusion

The present study was concerned with investigating the effect of topological interlocking of hard blocks on the mechanical performance of nacre-inspired materials. Our results demonstrate that by combining a biomimetic approach with the concept of topological interlocking, hybrid structures with superior mechanical properties can be obtained. The potency of the promising design proposed was validated by experimental results obtained under Mode I fracture testing (notched tensile testing). Additive manufacturing was employed as the enabling technology to create hybrid structures comprised by hard blocks with intricate interlocking surfaces interleaved with soft layers. The concavo-convex surfaces used in the interlocking osteomorphic blocks appear to be superior to the tablet-level waviness observed in nacre. It was demonstrated that topological interlocking opens up a new avenue to enhancing the mechanical performance of the composite structures by preventing the brittle failure through the stiff and brittle elements. Our results provided a clear demonstration that by introducing an interlocking-generating shape variation of the hard building blocks in the direction normal to the plane of assembly, further improvements in the mechanical properties of brick-and-mortar based structures are possible. Thus, the present work highlighted the additional benefits topological interlocking can bring to bio-inspired materials.

## Methods

Fabrication of all specimens was conducted using an Objet Connex500 multi-material 3D printer manufactured by Stratasys, Ltd. This machine employs polymer jetting technology which uses small nozzles to dispense liquid photopolymerisable monomer from a print head analogous to two-dimensional inkjet printing. This material is then cured by ultraviolet (UV) light using a source situated on-board of the printing head, which immediately causes the liquid resin to react and solidify. The material is forced through the printing unit, which contains eight printing heads, each containing 96 nozzles with 50 μm diameter. The nozzle size controls the accessible length scale of the 3D printer and plays a determinant role in the scale of the assemblies.

This particular technology employs a number of polymers, which are generally based upon proprietary acrylic-based, photopolymerisable monomers (a combination of various acrylics). Significantly, this technology is capable of combining rigid and rubbery polymers *in situ*, allowing for control of mechanical properties by mixing these polymers in various proportions. Two polymers in particular, VeroWhitePlus (VW+) and TangoBlackPlus (TB+), represent the two ends of the polymer spectrum. VW+ is a white, stiff/rigid polymer and TB+ is a black, soft rubber-like polymer. By using this technology, it is possible to manufacture parts composed of two highly varied base materials with near perfect interfaces. The widely differing values of the elastic modulus between these two materials makes them convenient candidates for synthesis of nacre-like composites. The mechanical response of these materials was reported previously, and our own testing (presented in the Supporting Information) agrees with this literature.

The specimens in the form of flat panels were printed with the soft phase comprising approximately 13.5% by volume, with an in-plane thickness of 250 μm. The dimensions of the samples were 64.75 mm × 58.5 mm and 3.125 mm in thickness. The specimens were fabricated with 10 elements (blocks) along the tensile direction and 24 elements across. The four block geometries presented in Fig. 1 had the same dimensions and the printed elements were of the same volume.

Mechanical characterization involved single edged notched tensile (Mode I) testing of the specimens in triplicate at a minimum for each geometry. The samples were printed with notches (9.745 mm by 0.79 mm), without the need for additional cutting prior to testing. Failure of the samples always occurred at the notch tip, which indicated that the printed notch was sufficiently sharp. The specimens were loaded in an Instron 4505 testing machine with a 20 kN static load cell. The samples were printed with specifically designed “knurled” VW+ sections at either end of the architectured sample region. The size and shape of these knurled sections were intended to minimise slippage with the machine’s grips when applying the tensile load. Testing was conducted with 3 mm/min displacement rate. It is important to note that these testing conditions did not follow any established standard; rather, the experimental procedure used by Dimas *et al*.^55^ in their study of brick-and-mortar structures was adopted. While the apparatus was designed to impart uniaxial tensile forces to the specimens, as the crack propagated, non-uniformity of load occurred, leading to an induced torque when the force was applied to the uncracked region of a specimen.

Computational work was undertaken using commercial ABAQUS/Explicit software. In simulating assemblies of hard blocks without soft layers, a general contact algorithm was used as a penalty method (a stiffness-based approach) to model the contact between the blocks. Load non-uniformities leading to a developed torque in the experimental procedure as crack propagation advanced were not included in the simulations. Mesh convergence studies were carried out to determine the necessary element size. Sufficient mesh density was found to accurately represent stresses with adequate resolution within a block at approx. 4600 elements per block. The Explicit solver was employed due to its capability to deliver a convergent solution for highly nonlinear systems with many complex contacts under transient loads. However, the assemblies were assumed to be under quasi-static loads, which represented a challenge to the solver’s efficiency. In order to accelerate the quasi-static simulations, the time step was reduced artificially. The issue with this method is that if the simulation speed is increased too much, inertial forces may falsify the predicted response. To evaluate this effect, the time step was reduced incrementally until the results began to diverge, and hence become unrepresentative. The ratio of the kinetic energy to the internal energy for the shortest time increment providing accurate results was then calculated to ascertain that the kinetic energy was sufficiently small (i.e. less than 1% of the internal energy)^63^. The model was also evaluated qualitatively and gauged by the physical testing to ensure accuracy. A loading time of 0.1 s in the simulations was found to be adequate. The parameters selected for the simulations were based on previous calculations conducted by our group, as well as several other groups, for assemblies with the same materials under different loading conditions, which were verified experimentally, both quantitatively and qualitatively^37^,^48^,^51^,^64^. The parameters were then re-assessed for viability under the current loading conditions presented in this work, and were found to meet the criterion used (i.e. a low ratio of kinetic energy to internal energy). The reported simulations intended to represent only the onset of crack propagation and were used to investigate the distribution of stresses around the crack tip and the adjacent blocks. The behaviour of the soft phase was modelled by setting the coefficient of friction between the blocks at a value of μ = 1.0, which emulates the ‘stickiness’ between the soft and hard phases. The obtained results have at least qualitative validity, as the material properties representing the contact behaviour were derived from experiment and the explicit solver variables did meet the quasi-static requirements^63^.

## Additional Information

**How to cite this article**: Djumas, L. *et al*. Enhanced Mechanical Performance of Bio-Inspired Hybrid Structures Utilising Topological Interlocking Geometry. *Sci. Rep.* **6**, 26706; doi: 10.1038/srep26706 (2016).

## Supplementary Material

Supplementary Information
