# Supplementary material for: Enhanced Mechanical Performance of Bio-Inspired Hybrid Structures Utilising Topological Interlocking Geometry
Source: Sci Rep. 2016 May 24;6:26706. doi: 10.1038/srep26706 (PMC4877644; doi:10.1038/srep26706)
Supplement: Supplementary Information [file srep26706-s1.doc]

Supplementary Information

# **Enhanced mechanical performance of bio-inspired hybrid structures utilising topological interlocking geometry**

*Lee Djumas*, Andrey Molotnikov, George P. Simon, Yuri Estrin*

**Material Properties**

The base materials used in 3D printing were tested under standard uniaxial tensile conditions (ASTM D638). This testing was primarily to determine mechanical properties (Young’s modulus and Poisson’s ratio) for computational work as well as to verify previously reported data.^[55]^ Additionally, testing of VW+ and TB+ was conducted following the same procedure used for composite samples, as outlined in the Experimental Section. This was used for comparison to the work conducted by Dimas,^[48]^ and good agreement was found. Some differences in the results can be attributed to small variations in experimental conditions, in particular in the method for notching samples that was different in the two studies. The results of the mechanical tests used to identify material parameters are presented in Figs. S1-S4. The supplementary videos show representative fracture testing of the four geometries investigated in this study; osteomorphic, brick-and-mortar, hourglass and smooth honeycomb. Additionally, a representative simulation is shown of the hourglass geometry.

**Figure S1.** Stress-strain plot of VW+ tested under notched mode I tensile test conditions as described in Experimental Section**Figure S2.** Stress-strain plot of TB+ tested under notched mode I tensile test conditions as described in Experimental Section

**Figure S3.** Stress-strain plot of VW+ tested under uniaxial tensile test conditions

**Figure S4.** Stress-strain plot of TB+ tested under uniaxial tensile test conditions
